# 220–325 GHz all-photopolymer Bragg horn antennas towards eco-friendly terahertz applications

**DOI:** 10.1038/s41598-025-11978-9

**Published:** 2025-07-30

**Authors:** Nonchanutt Chudpooti, Binbin Hong, Rungrat Viratikul, Feaveya Kheawprae, Akkarat Boonpoonga, Panuwat Janpugdee, Weijia Zhang, Prayoot Akkaraekthalin, Joachim Oberhammer, Nutapong Somjit

**Affiliations:** 1 Faculty of Applied Science, King Mongkut’s University of Technology North Bangkok, Bangkok, 10800 Thailand; 2 Faculty of Arts and Sciences, Beijing Normal University, Zhuhai, 519085 China; 3 Department of Electrical Engineering, Faculty of Engineering, Chulalongkorn University, Bangkok, 10330 Thailand; 4 Faculty of Engineering and Technology, King Mongkut’s University of Technology North Bangkok (Rayong Campus), Rayong, 21120 Thailand; 5 Department of Electrical and Computer Engineering, Faculty of Engineering, King Mongkut’s University of Technology North Bangkok, Bangkok, 10800 Thailand; 6 Department of Mathematical and Information Sciences, Shaoxing University, Shaoxing, 312000 China; 7 Institute of Artificial Intelligence, Shaoxing University, Shaoxing, 312000 China; 8 Key Laboratory of Artificial Intelligence Multi-Dimensional Application Research, Shaoxing, 312000 China; 9 Department of Atmospheric, Oceanic and Planetary Physics, University of Oxford, Oxford, OX1 4BH UK; 10 Division of Micro and Nanosystems (MST), KTH Royal Institute of Technology, 100 44 Stockholm, Sweden; 11 School of Electronic and Electrical Engineering, University of Leeds, Leeds, LS2 9JT UK

**Keywords:** All-photopolymer Horn antenna, Bragg structure, THz antennas, Engineering, Electrical and electronic engineering

## Abstract

This paper presents the development of the world’s first high-gain, all-photopolymer Bragg horn antennas explicitly designed for the WR-3.4 band (220–325 GHz), marking a groundbreaking advancement in terahertz (THz) antenna technology. Unlike conventional metallic horn antennas, which suffer from conductor losses and manufacturing complexity, this innovative design utilizes eco-friendly photopolymer materials and additive manufacturing, achieving a fractional bandwidth of 38.5% that fully covers the WR-3.4 band. The proposed antenna achieves a measured peak gain of 28.98 dBi at 300 GHz, with a return loss better than − 20dB across the band and a consistent half-power beamwidth (HPBW) of ~ 5°, ensuring precise directivity and minimal sidelobe interference. By employing a novel horn-type adapter for seamless mode conversion from TE_10_ to the fundamental HE_11_ mode, the design significantly enhances coupling efficiency and reduces signal loss. Additionally, fabrication costs can be reduced by over 50% compared to traditional metallic designs, while maintaining repeatability and enabling rapid prototyping. As the first demonstration of photopolymer-based antennas achieving such high gains in the 220–325 GHz THz spectrum, this work establishes a new benchmark in THz antenna technology, providing an eco-friendly, cost-effective, and high-performance solution for high-speed communication, medical diagnostics, security imaging, and spectroscopy applications.

## Introduction

Recently, terahertz (THz) technology has attracted significant attention from researchers and engineers worldwide due to its vast potential across various applications, including high-resolution radar systems, imaging, sensing, security scanning, and high-speed communication networks^1–10^. Antennas play a critical role in realizing these applications. Over the years, numerous THz antenna designs have been proposed, such as horn antennas, slotted waveguide antennas, reflector antennas, and dielectric lens antennas^11–18^. Advances in terahertz artificial microstructures have significantly expanded the degrees of freedom and efficiency for wave propagation manipulation^19–22^. Among these, dielectric lens antennas stand out for THz applications owing to their straightforward design, high gain, broad bandwidth, immunity to conductor losses, and ability to support circular polarization^23–35^. However, achieving efficient operation in the 220–325 GHz frequency range remains challenging. Traditional metallic horn antennas, optimized for lower frequencies, struggle to effectively handle THz radiation due to wavelength disparities. This impedance mismatch results in suboptimal signal transmission and reception, ultimately constraining the performance of THz technology.

In comparison to conventional metallic horn antennas, all-photopolymer Bragg Horn Antennas offer numerous advantages within the THz spectrum. These antennas demonstrate exceptional efficiency in signal transmission and reception, significantly enhancing the performance of THz technology. Their intrinsic miniaturization capability facilitates seamless integration into compact THz devices and systems, aligning with the growing demand for smaller and more efficient components in THz technology. Furthermore, these antennas provide precise control over radiation patterns, making them highly adaptable for diverse THz applications, including high-speed wireless communication, advanced THz imaging for medical diagnostics and security scanning, and precise spectroscopy for chemical analysis. Notably, their material properties are meticulously optimized to ensure superior performance across the THz frequency range.

Several dielectric-based and hybrid horn antennas have been proposed to overcome the limitations of traditional metallic designs at terahertz frequencies. For instance, a multiflare horn designed to operate at 1.9 THz was developed for spaceborne instrumentation^36^. Surface polishing techniques to reduce roughness—which critically affects antenna performance at THz frequencies—were demonstrated by Montofre et al. in the 211–275 GHz range^37^. At these high frequencies, the short wavelength significantly impacts antenna dimensions, making fabrication particularly challenging. This is especially true for small antennas and their feeding networks, which demand high-precision machining and experienced human operation. In^38^, a 3D-printed back-to-back horn antenna was presented for life sciences applications, showcasing the potential of additive manufacturing in THz systems. A free-space THz measurement setup incorporating 3D-printed hemispherical lens antennas for both transmitter and receiver modules was introduced in^39^. Further innovations include high-gain horn antennas, corrugated H-plane horns, and implementations with and without LTCC integration, as reported in^40–44^. While these designs represent significant progress, they often depend on complex metallic or hybrid fabrication processes. In contrast, the approach presented in this paper introduces a fully photopolymer-based Bragg horn antenna fabricated using additive manufacturing, offering a simplified, low-cost solution suitable for integration into next-generation THz systems.

All-photopolymer Bragg horn antennas offer several noteworthy advantages. They excel in repeatability and accuracy, ensuring consistent and precise results in terahertz measurements. Additionally, their user-friendly design simplifies setup and operation, reducing the complexity of measurement procedures and making them accessible to researchers and technicians. These antennas are also cost-effective, providing an economical solution for THz measurements compared to many conventional techniques. Their inherent miniaturization capabilities enable seamless integration into compact THz devices and systems, aligning with the ongoing trend toward miniaturization in THz technology and fostering the development of portable and efficient THz solutions. Notably, their ability to provide precise control over radiation patterns enhances their versatility, making them suitable for a wide range of THz applications.

In^11^, an all-dielectric terahertz horn antenna based on a hollow-core electromagnetic crystal structure (EMXT) was presented. The antenna operates in the frequency range of 100–190 GHz, with a reflection coefficient (S_11_) lower than − 30 dB. To optimize the horn antenna, key parameters such as the flare length and flare angle were investigated through parameter sweeps, allowing performance comparisons. This design employed a time-domain spectroscopy (TDS) transmitter as the feeding network. However, using a TDS transmitter presents practical challenges, as it requires experienced personnel for precise setup and calibration, which may be difficult for beginners. Furthermore, the high-precision alignment necessary for this antenna demands specialized scales and precision tools, potentially increasing setup complexity and cost.

This paper provides a comprehensive exploration of the 220–325 GHz all-photopolymer Bragg horn antennas, presenting a novel approach to advancing THz antenna technology. It examines the underlying principles, design considerations, and advantages of this innovative technology. By integrating theoretical analysis, numerical simulations, and experimental validation, the study demonstrates the ability of these antennas to deliver high-performance radiation characteristics within the challenging THz frequency range. The proposed design holds significant promise for a wide range of applications, including high-speed wireless communication, advanced THz imaging for medical diagnostics and security scanning, precise spectroscopy for chemical analysis, and improved quality control processes across various industrial sectors.

The distinctiveness of this research lies in its dedicated focus on the 220–325 GHz frequency range within the THz spectrum, a band that remains underrepresented in existing technologies. By leveraging all-photopolymer materials instead of traditional metallic horn antennas, this work introduces groundbreaking innovations. These advancements result in improved efficiency, enhanced miniaturization capabilities, precise radiation control, and optimized material properties tailored specifically for the challenging demands of the THz spectrum. This targeted and innovative approach paves the way for exploring new applications in high-speed communication, advanced THz imaging, precise chemical analysis, and improved quality control across diverse industries.

In addition to their numerous advantages, these antennas exhibit material properties specifically optimized for operation within the THz frequency range. These optimizations include favorable permittivity and permeability characteristics, which significantly enhance antenna performance and establish a clear advantage over traditional metallic horn antennas. However, it is important to note that while all-photopolymer Bragg horn antennas deliver exceptional performance in the millimeter-wave (mmWave) and THz frequency ranges, their applicability at lower frequencies is inherently limited. This limitation arises from their precisely engineered design, which is tailored to the shorter wavelengths characteristic of the THz spectrum.

## Design and fabrication of all-photopolymer Bragg Horn antennas

### Design of all-photopolymer Bragg Horn antennas

**Fig. 1:**
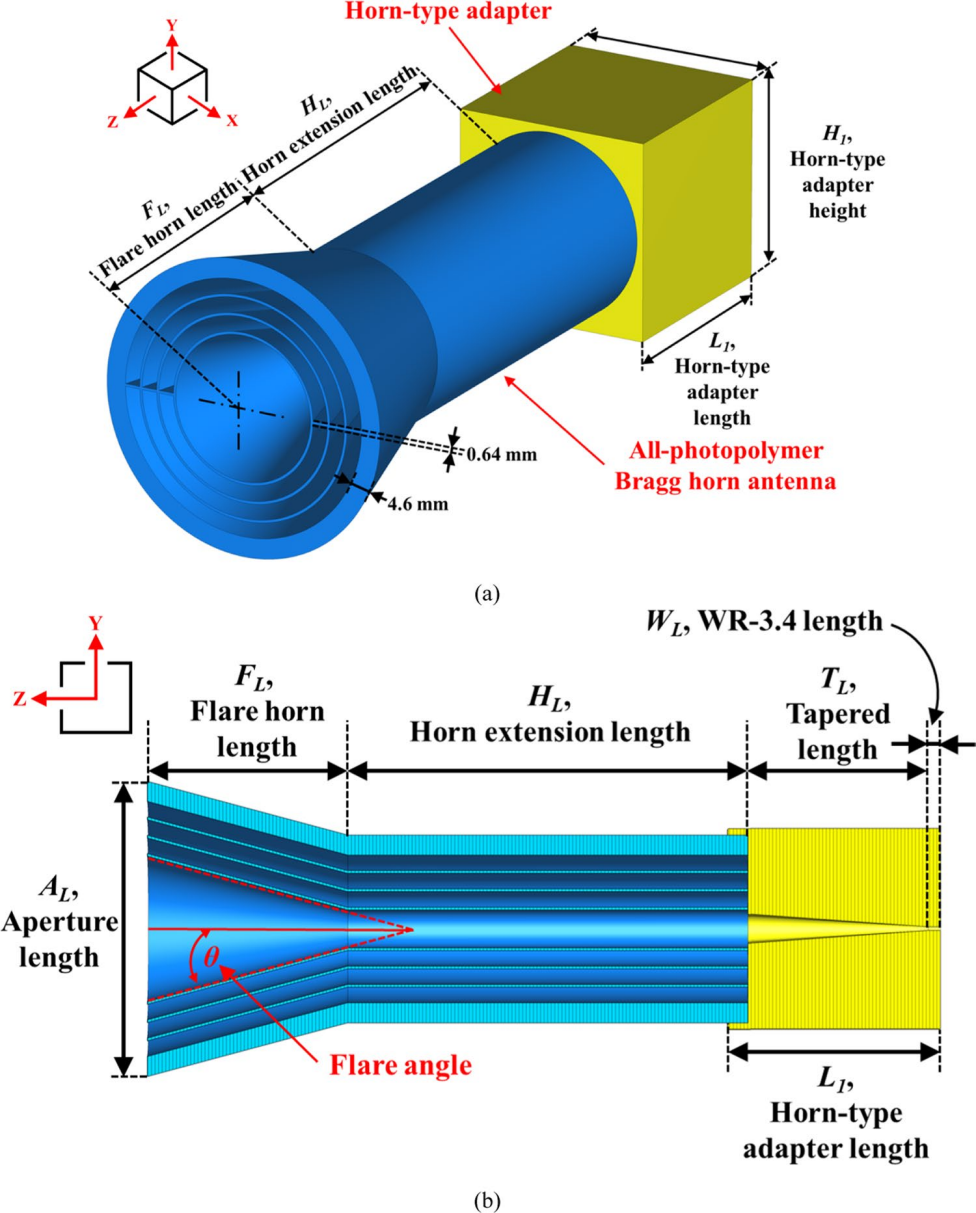
The geometry of the 220–325 GHz all-photopolymer Bragg horn antenna, consisting of two components: the horn-type adapter and the all-photopolymer Bragg horn antenna (**a**) perspective view and (**b**) The cross-sectional view in *YZ*-plane.

All-photopolymer Bragg horn antennas, fabricated from Accura ClearVue (experimentally characterized refractive index $\:n\left(f\right)=-0.0123{f}^{2}-0.0335f+1.6262$ and absorption coefficient $\:\alpha\left(f\right)=6.4667{f}^{2}+17.5066f-2.0294$ over 0.2–1 THz^33^), are specialized devices designed for operation in the terahertz (THz) frequency range, specifically between 220 GHz and 325 GHz. These antennas stand out due to their non-metallic construction, which makes them highly suitable for THz applications. Their unique horn-shaped design plays a critical role in precisely controlling signal emission, facilitating their use in various advanced fields. Figure 1(a) illustrates the perspective geometry of the Bragg fiber horn antenna integrated with the horn-type adapter^33^. Within the Bragg fiber structure, the outermost layer—measuring 4.6 mm in thickness with a support bridge width of 0.64 mm—serves as a robust protective polymer layer. This layer enhances mechanical stability, provides resistance to environmental conditions, absorbs residual electromagnetic waves, and effectively shields the fiber from external interference^33^.

**Table 1:** The key parameters of the all-photopolymer Bragg Horn antennas and Horn-type Adapter.

Parameter	Description	Value
*A*_*L*_	Aperture length	55.60, 73.65, 104.58 mm
*F*_*L*_	Flare horn length	50 mm
*H*_*L*_	Horn extension length	100 mm
*θ*	Flare angle	5, 15, 30 degrees
*W*_*L*_	WR-3.4 aperture length	3 mm
*T*_*L*_	Tapered length	45.3 mm
*L*_*1*_	Horn-type adapter length	53 mm
*W*_*1*_	Horn-type adapter width	50 mm
*H*_*1*_	Horn-type adapter height	50 mm

The horn-type adapter serves as a critical component, connecting a standard WR-3.4 waveguide to a Bragg fiber through its input aperture. It performs mode conversion from the TE₁₀ mode in the rectangular waveguide to the fundamental HE₁₁ mode in the Bragg fiber, ensuring efficient coupling and minimizing reflection losses. It facilitates the conversion of the TE_10_ mode from the rectangular waveguide into the fundamental HE_11_ mode within the Bragg fiber, which is characterized by linear polarization^33^. Unlike the input mode described in^34^, which corresponds to a free-space Gaussian beam, the mode in this work is a circular waveguide mode at the output aperture of the horn-type adapter. This hybrid mode consists of multiple competing components, with the fundamental TE_11_ mode of the hollow metallic circular waveguide (HMCW) being the primary mode. Achieving proper phase matching is essential for minimizing signal loss and reflection, thereby ensuring efficient electromagnetic energy transfer between the waveguides. Effective phase matching allows seamless energy propagation from the horn-type adapter to the THz Bragg fiber, significantly enhancing measurement accuracy and reliability.

In this work, the TE_10_ mode in the WR-3.4 rectangular waveguide is converted to the TE_11_ mode in the hollow metallic circular waveguide (HMCW) by the horn-type adapter while maintaining vertical polarization and is then further transformed into the HE_11_ mode within the Bragg fiber. This mode exhibits low attenuation in dielectric waveguides, Gaussian-like field distribution, and efficient coupling characteristics beneficial for terahertz system applications. The transmission coefficient (S_21_) of the adapter is lower than 1 dB across the 220–325 GHz band, confirming minimal energy loss during mode transitions and ensuring effective mode conversion for the hybrid structure. These details align with the results reported in^33^.

Figure 1(b) provides a cross-sectional view of the all-photopolymer Bragg horn antenna, with the key parameters of both the Bragg fiber horn and the horn-type adapter summarized in Table 1.

The design of the Bragg fiber horn antenna is guided by electromagnetic wave propagation principles in dielectric waveguides, hybrid mode transformation, and impedance tapering techniques. The structure supports the propagation of the fundamental HE_11_ mode, a hybrid mode analogous to the LP_01_ mode in optical fibers, which can be efficiently excited via a carefully designed horn transition.

The radial confinement mechanism in the Bragg fiber portion relies on the photonic bandgap effect formed by periodic changes in dielectric impedance. This behavior can be understood using a simplified Bragg reflection condition, where constructive interference occurs for waves reflected at dielectric interfaces:

1 $$2 \cdot {n_{eff}} \cdot d\cos \theta =m\lambda$$

where *n*_*eff*_ is the effective refractive index, *d* is the thickness of the dielectric layer, *θ* is the propagation angle within the fiber, λ is the wavelength in free space, and *m* is an integer indicating the Bragg order. For the horn region, the aperture gain and directivity are approximated using classical horn antenna theory:

2 $$G=\eta \left( {\frac{{4\pi A}}{{{\lambda ^2}}}} \right)$$

where *G* is the gain, *η* is the radiation efficiency, *A* is the aperture area, and λ is the free-space wavelength. This relationship guides the optimization of the flare angle (θ) and aperture length (*A*_*L*_) to balance gain, beamwidth, and structural compactness.

The Bragg fiber horn antenna is defined by three interdependent geometric parameters: the flare angle (θ), the aperture length (*A*_*L*_), and the flare horn length (*F*_*L*_). Due to the geometric dependency between flare angle and aperture size, this study focuses on varying the flare angle while keeping the horn length fixed. Specifically, *F*_*L*_ is set to 50 mm, and the flare angle is swept from 5° to 30°, which defines the aperture via the relation:

3 $${A_L}=2 \cdot {F_L} \cdot \tan \theta$$

This configuration allows control over the beam collimation and impedance transition characteristics. To evaluate the antenna’s performance, parametric simulations were conducted across different flare angles to assess key metrics such as reflection coefficient (S_11_), realized gain, and far-field radiation behavior at boresight (θ = 0°).

In this study, the CST Studio Suite^35^ was utilized to optimize the design of the all-photopolymer Bragg horn antenna. The optimization aimed to maximize the realized gain while maintaining a return loss below − 20 dB across the 220–325 GHz band. The flare angle (θ) was selected as the primary variable, and parametric sweeps were conducted to determine the optimal configuration. The horn extension length was fixed at 100 mm to facilitate the transition of the electromagnetic field into the fundamental HE_11_ mode within the Bragg fiber^34^. Additionally, the flare horn length (*F*_*L*_) was set at 50 mm to minimize insertion loss in the hollow Bragg fiber. As a result, the flare angle (θ) was identified as the primary variable for optimizing the gain performance of the Bragg fiber antenna.

**Fig. 2:**
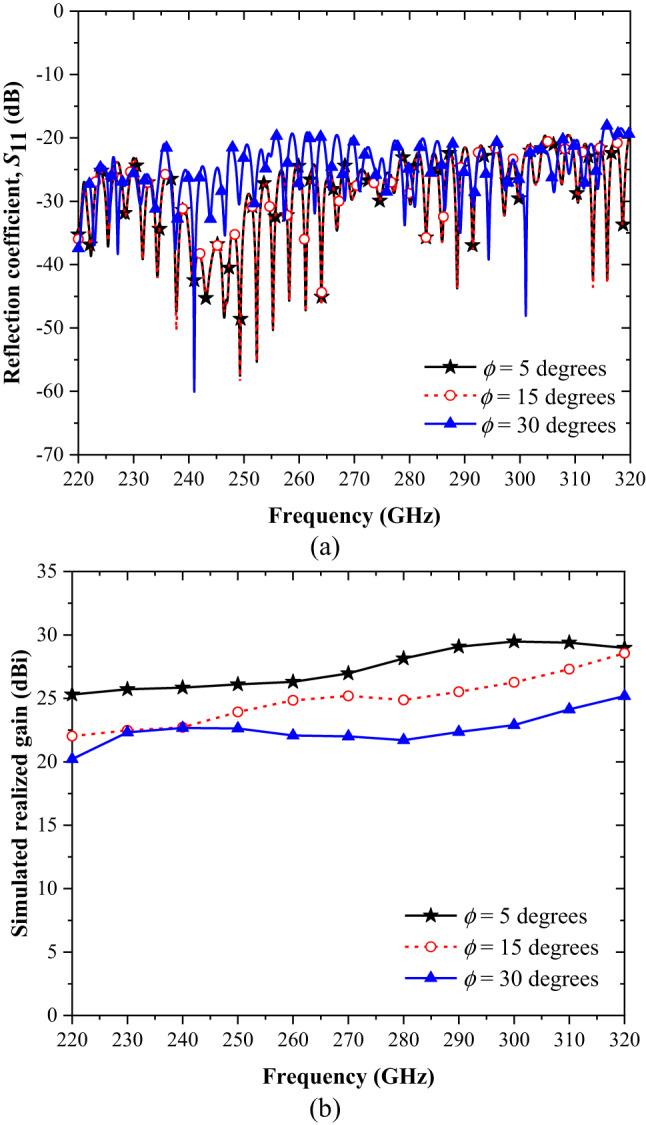
Simulated parametric study of the flare angle, *θ*, for (**a**) reflection coefficient, *S*_11_, and (**b**) realized gain of antenna at 0 degree.

Figure 2 illustrates the simulated results for varying the flare angle from 5° to 30°. As shown in Fig. 2(a), the reflection coefficient (S_11_) across the full operating band of 220–325 GHz is slightly higher for a flare angle of 30° compared to 5° and 15°. Despite this, the antenna exhibits favorable performance, maintaining S_11_ values below − 20 dB across the entire band. Figure 2(b) presents the simulated realized gain of the Bragg fiber horn antenna at 0° for the three flare angles: 5°, 15°, and 30°. The results reveal that all configurations achieve a realized gain exceeding 20 dBi across the band, with the 5° flare angle delivering the highest performance. For the 5° configuration, the realized gain surpasses 25.3 dBi throughout the band, peaking at 29.48 dBi at 300 GHz.

**Fig. 3:**
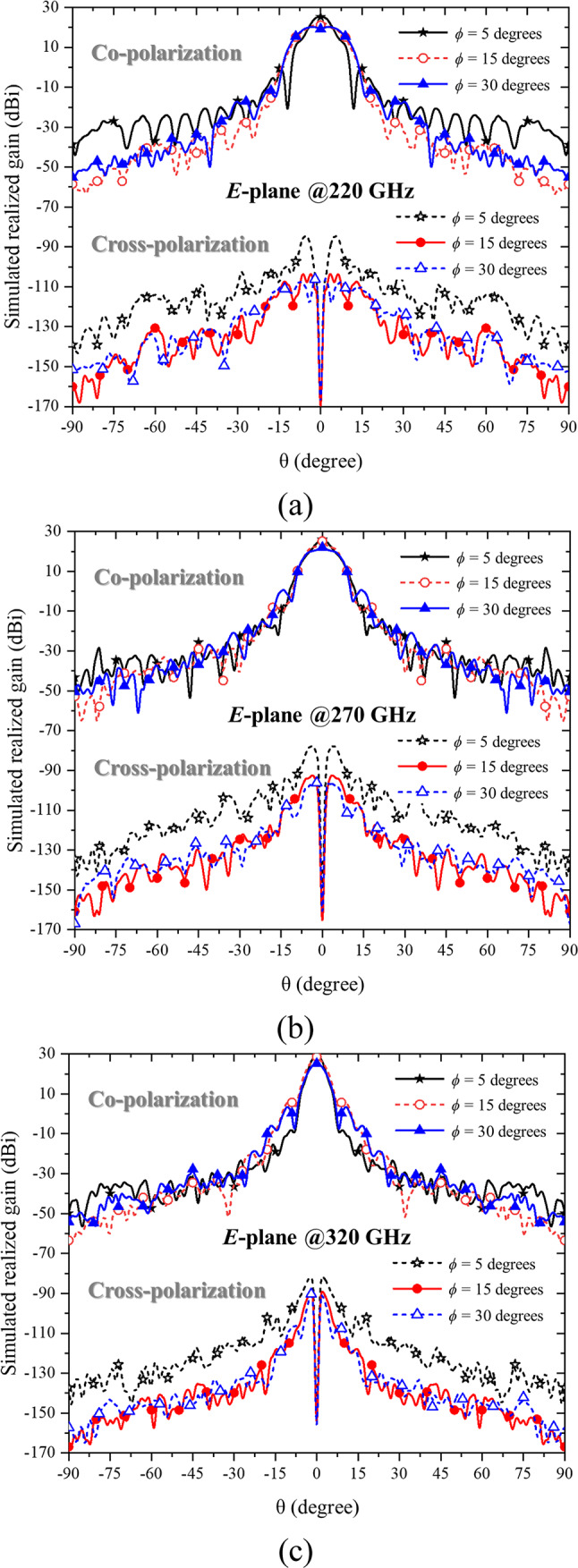
The simulated 2D *E*-plane radiation patterns showing co-polarization and cross-polarization components for three flare angle (*θ*) configurations of 5 degrees, 15 degrees, and 30 degrees at (**a**) 220 GHz, (**b**) 270 GHz, and (**c**) 320 GHz.

**Fig. 4:**
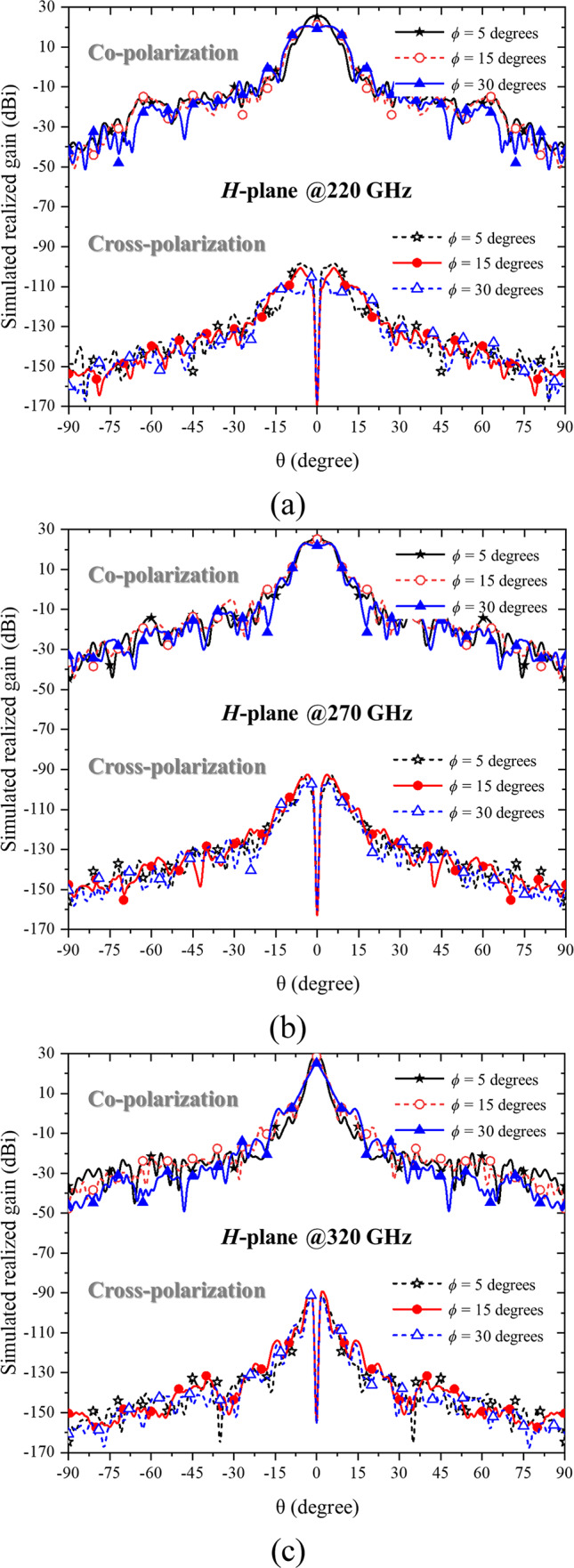
The simulated 2D *H*-plane radiation patterns showing co-polarization and cross-polarization components for three flare angle (*θ*) configurations of 5 degrees, 15 degrees, and 30 degrees at (**a**) 220 GHz, (**b**) 270 GHz, and (**c**) 320 GHz.

Based on the radiation pattern plots, the angular definitions adhere to the standard spherical coordinate system employed in CST Microwave Studio. Theta represents the elevation angle measured from the antenna’s main axis (bore-sight), while phi corresponds to the azimuthal angle around that axis. To illustrate the antenna’s radiation characteristics in both principal planes, Fig. 3 presents the *E*-plane patterns (phi = 0°), and Fig. 4 presents the *H*-plane patterns (phi = 90°). These figures include both co-polarization and cross-polarization results across the three flare angles (5°, 15°, and 30°).

Figure 3 depicts the *E*-plane radiation patterns at three sampling frequencies—220 GHz, 270 GHz, and 320 GHz—representing the lower, middle, and upper frequencies of the WR-3.4 band, respectively. At 220 GHz, the maximum gains achieved for the three flare angles (5°, 15°, and 30°) are 25.3 dBi, 22.0 dBi, and 20.2 dBi, with corresponding half-power beamwidths (HPBWs) of 6.7°, 10.9°, and 15.4°. At the center frequency of 270 GHz, the maximum gains for the same flare angles are 26.0 dBi, 25.2 dBi, and 22.0 dBi, with HPBWs of 5.6°, 6.3°, and 11.1°, respectively. At 320 GHz, representing the upper end of the operating band, the maximum gains for the three configurations are 29.0 dBi, 28.6 dBi, and 25.2 dBi, with corresponding HPBWs of 4.3°, 4.5°, and 5.2°.

Figure 4 illustrates the *H*-plane radiation patterns corresponding to the same sampling frequencies of 220 GHz, 270 GHz, and 320 GHz, covering the full WR-3.4 band. Similar to the *E*-plane results, the antenna demonstrates strong directional performance across all flare angles. At 220 GHz, the recorded peak gains are 25.5 dBi, 21.5 dBi, and 20.6 dBi for flare angles of 5°, 15°, and 30°, respectively, accompanied by HPBWs of 8°, 15.2°, and 15.9°. At 270 GHz, the gains rise to 26.5 dBi, 25.2 dBi, and 23.2 dBi, with beamwidths narrowing to 7.9°, 9.5°, and 13.4°. At the upper frequency of 320 GHz, the maximum gains increase further to 29.0 dBi, 28.6 dBi, and 25.2 dBi, with corresponding HPBWs of 4.3°, 2.7°, and 3.2°. These results confirm that the *H*-plane patterns follow trends similar to the *E*-plane, demonstrating consistent gain behavior and beam symmetry, which reinforce the effectiveness of the Bragg fiber horn design across both principal planes.

The simulation results for the three evaluated parameters—reflection coefficient (S_11_), realized gain at 0°, and radiation patterns across flare angles of 5°, 15°, and 30°—indicate that the Bragg fiber horn antenna with a 5° flare angle delivers the best overall performance under all conditions. This configuration consistently outperforms the other flare angles in terms of gain, efficiency, and beamwidth, making it the optimal choice for the studied frequency range. Despite the limitation in acquiring measured cross-polarization data, the simulated cross-polarization levels remain below − 35 dB across the main lobe in both the *E*- and *H*-planes, and the radiation patterns exhibit strong symmetry, as demonstrated in Figs. 3 and 4.

### Fabrication of all-photopolymer Bragg Horn antennas

The fabrication process of the Bragg fiber horn antenna began with its design, which was meticulously developed using CST simulation software to optimize performance parameters.

**Fig. 5:**
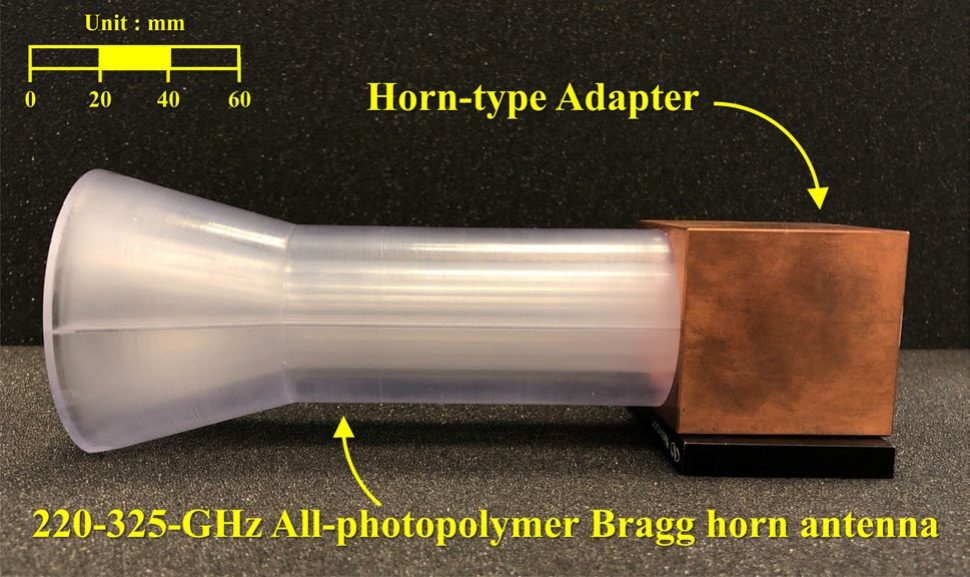
The fabricated 220–325 GHz all-photopolymer Bragg horn antenna with a flare angle of 5 degrees, integrated with the horn-type adapter.

The finalized design was then implemented using a 3D Systems PolyJet 7000 HD 3D printer, leveraging the stereolithography (SLA) technique to achieve high precision in manufacturing the all-photopolymer structure, with a percentage-based tolerance of 0.1% (approximately 0.025 mm per 25.4 mm). The 3D printing process for a single Bragg horn antenna required approximately 27 h. Following this, the horn-type adapter was fabricated using CNC milling on a Jingdiao JDHGT600T machine, which offers a positioning accuracy of 2 μm. The CNC milling process for each copper connector took approximately 20 h to complete, ensuring excellent dimensional accuracy and surface quality. To ensure stable alignment during assembly, the horn-type adapter was designed with a notch structure that securely locks with the 220–325 GHz all-photopolymer Bragg horn antenna. On the opposite side, the adapter connects to the WR-3.4 waveguide using a screw-based locking mechanism designed to the WR-3.4 standard, ensuring reliable positioning during measurement and minimizing alignment errors. This mechanical locking approach facilitates repeatable measurement setup while protecting the integrity of the photopolymer structure during handling. A copper connector was specifically flared to match the diameter of the Bragg fiber, ensuring proper alignment and minimal signal loss. The CNC milling process provided the precision necessary to meet the design specifications and facilitated seamless integration of the adapter with the Bragg fiber horn antenna. The final assembly, depicted in Fig. 5, features an all-photopolymer Bragg horn antenna optimized for the 220–325 GHz frequency range. The design includes a flare angle of 5°, which was selected for its superior performance in simulations, and integrates efficiently with the horn-type adapter to enhance overall functionality.

Key fabrication challenges included slight warping in thin-wall polymer regions, tight alignment tolerances at the metal–polymer interface, and long processing times. The copper adapter, while precisely milled with 2 μm accuracy, required additional care due to surface roughness and fragility of thin metallic walls. Future improvements may include integrated alignment features and optimization of material properties to reduce dielectric loss.

## Measurement results

To evaluate the performance of the all-photopolymer Bragg horn antenna, measurements were conducted using a Keysight Technologies PNA-X N5242 Vector Network Analyzer (VNA), equipped with two OML WR-3.4 frequency extender heads. These measurements included the reflection coefficient (S_11_) and radiation pattern over the 220–325 GHz frequency range. The Line-Reflect-Line (LRL) calibration technique was employed to ensure accurate and reliable data across the operating band. A laser alignment system, commonly utilized in terahertz (THz) measurements, was implemented to achieve precise alignment and controlled rotation of the antenna during testing, minimizing alignment errors and enhancing measurement accuracy. A standard WR-3.4 conical horn antenna^45‚46^ was used as the transmitting reference, providing a well-characterized and repeatable radiation pattern for comparative analysis. All measurements were conducted in a temperature-controlled laboratory environment, maintained at 25 ± 2 °C, with relative humidity below 60%, typically ranging from 45 to 55%. These conditions help reduce the effects of environmental variation on signal propagation and measurement repeatability.

Several potential sources of measurement error were considered and mitigated. Misalignment was addressed through the use of a laser alignment system to precisely position and rotate the antenna under test, with repeated alignment checks verifying position accuracy. The alignment error due to the rigidity difference between the photopolymer antenna and the metal waveguide port was found to be within approximately 0.2 millimetesrs in lateral displacement, which may result from minor photopolymer shrinkage or expansion. The measurement environment was controlled to minimize temperature variations during setup, ensuring stable conditions and reducing thermal expansion effects. This minor alignment deviation did not significantly affect the measured gain or radiation patterns throughout the experiments. Connector repeatability was ensured by using high-quality WR-3.4 flanges with torque-controlled screws. Free-space path loss and external reflections were minimized by surrounding the measurement area with terahertz-absorbing materials and maintaining a 50 cm line-of-sight distance between the transmitter and AUT. This distance satisfies the far-field condition of the standard horn and exceeds the reactive near-field boundary of the proposed Bragg horn antenna. These measures collectively enhance the accuracy and credibility of the reported gain and radiation pattern results.

**Fig. 6:**
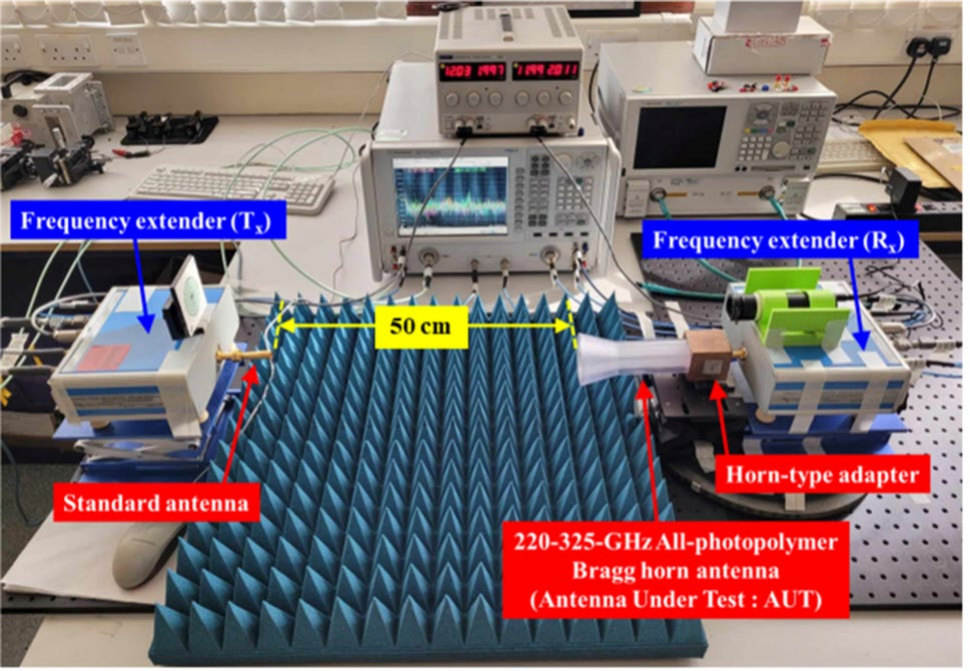
Measurement setup used in this study, comprising a standard conical horn antenna in the transmitter section (T_x_) and the all-photopolymer Bragg horn antenna in the receiver section (R_x_), serving as the antenna under test (AUT).

The measurement setup, illustrated in Fig. 6, consists of a standard conical horn antenna connected to a WR-3.4 frequency extender acting as the transmitter (Tx) and the all-photopolymer Bragg horn antenna, integrated with a horn-type adapter and WR-3.4 frequency extender, serving as the receiver (Rx) and antenna under test (AUT). This configuration ensured a controlled and repeatable environment for assessing the antenna’s performance metrics.

**Fig. 7:**
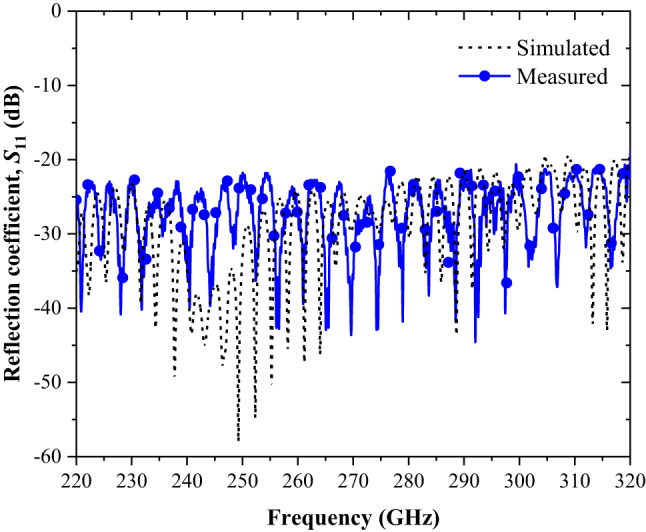
The comparison results of simulated and measured reflection coefficient, S_11_ for the whole WR-3.4 band (220–325 GHz).

Figure 7 compares the measured and simulated results for the reflection coefficient (S_11_) across the full WR-3.4 frequency band (220–325 GHz). The data confirm that the antenna design achieves excellent performance, maintaining an S_11_ value consistently below − 20 dB across the entire band. This indicates minimal signal reflection and efficient energy transfer. The results also highlight a fractional bandwidth of approximately 38.5%, underscoring the antenna’s capability to operate effectively over a broad frequency range while meeting the stringent requirements of THz applications.

**Fig. 8:**
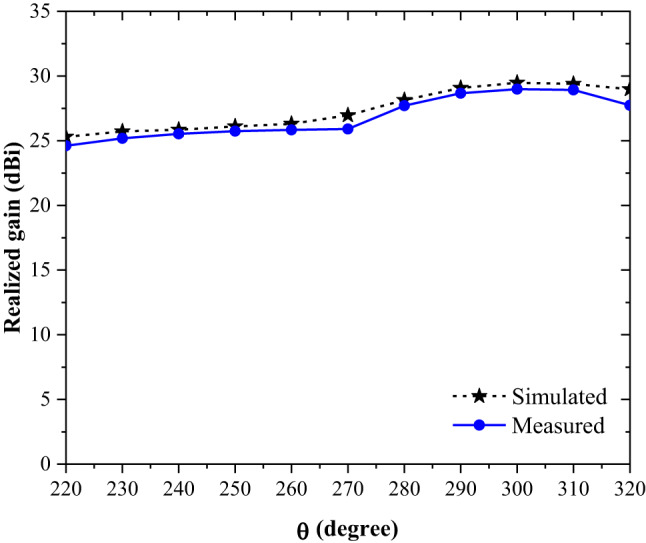
The comparison results of simulated and measured realized gain at 0 degree for the whole WR-3.4 band (220–325 GHz).

The broadband gain of the proposed all-photopolymer Bragg horn antenna was determined by recording the transmission coefficients (S_21_) and using them to calculate the realized gain in units of dBi. Figure 8 illustrates the comparison between the simulated and measured realized gain of the antenna across the 220–320 GHz frequency range, with data collected at 10 GHz intervals. Table 2 provides the detailed results, including the calculated percentage differences between the simulated and measured values. The findings indicate that the maximum error between the simulated and measured gain is within 4.28%, demonstrating the high accuracy and reliability of the measurement system employed in this study. These results validate the robustness of the design and the effectiveness of the testing methodology.

**Table 2:** The calculated comparison results of simulated and measured realized gain at 0 degree.

Frequency (GHz)	Simulated realized gain (dBi)	Measured realized gain (dBi)	Error (%)
220	25.31	24.61	2.77
230	25.72	25.19	2.06
240	25.85	25.53	1.24
250	26.10	25.74	1.38
260	26.31	25.83	1.82
270	26.67	25.91	2.85
280	28.14	27.72	1.49
290	29.07	28.67	1.38
300	29.48	28.98	1.70
310	29.39	28.92	1.60
320	28.98	27.74	4.28

**Fig. 9:**
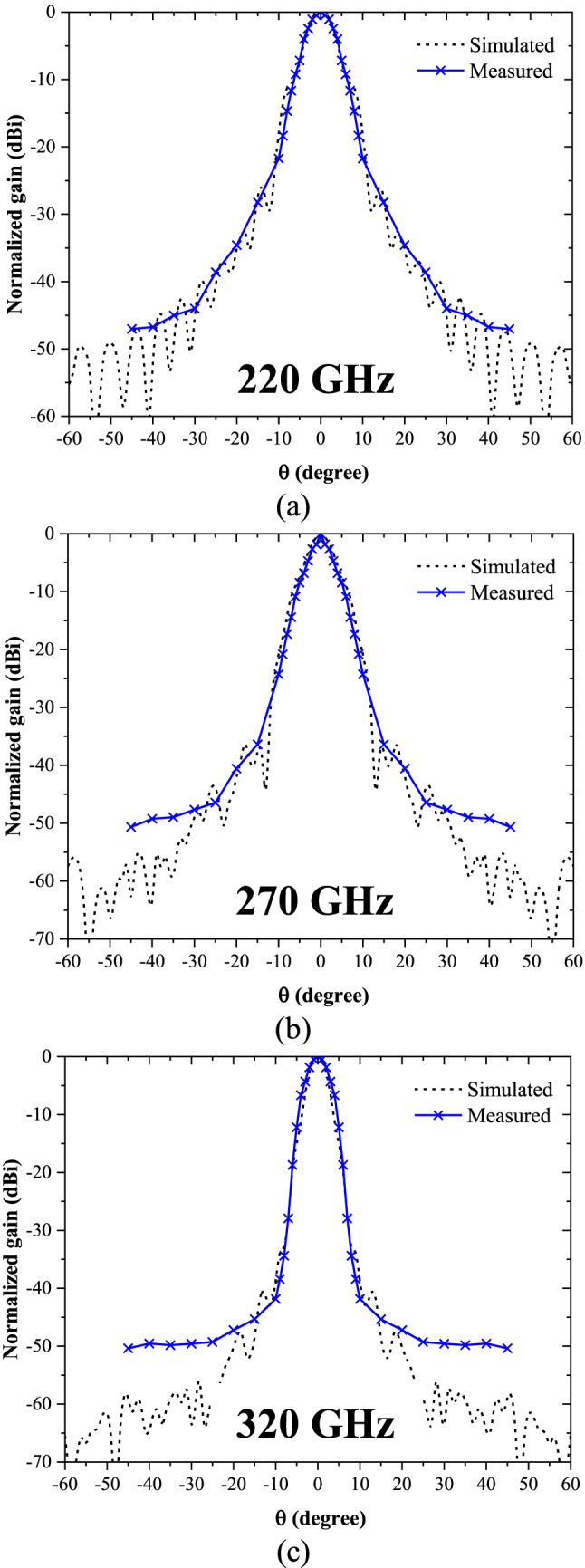
The simulated and measured radiation patterns for flare angle () configurations of 5 degrees at (**a**) 220 GHz, (**b**) 270 GHz, and (**c**) 320 GHz.

For radiation pattern measurements, a WR-3.4 horn antenna with a nominal gain of 26 dBi and a half-power beamwidth (HPBW) of 10° was utilized as the standard reference antenna. The radiation characteristics of the all-photopolymer Bragg horn antenna were measured using a Keysight VNA equipped with frequency extender heads. Precise antenna positioning and rotation during the measurements were ensured using a laser alignment system, which minimized alignment errors and enhanced measurement accuracy. The test setup maintained a 50 cm separation between the antenna under test (AUT) and the reference antenna, a distance exceeding the simulated far-field requirement for the upper frequency of 320 GHz. The AUT was manually rotated and adjusted across a range of angles from − 45° to + 45°. Within the central range of − 10° to + 10°, 1° increments were used, while 5° increments were applied for the wider range of ± 10° to ± 45°. This controlled rotation ensured comprehensive angular coverage for the evaluation. The radiation pattern of the Bragg fiber horn antenna was measured at three representative frequencies—220 GHz, 270 GHz, and 320 GHz—corresponding to the lower, middle, and upper ranges of the WR-3.4 band (220–325 GHz). Figure 9 (a)–(c) illustrates the normalized comparison between the measured and simulated radiation patterns of the antenna at these frequencies, highlighting the performance and agreement between the two datasets.

**Table 3:** Comparisons of key properties between different THz antennas.

Ref. No.	Antenna Structure	Operating Band (THz)	Overall size W×L×H (mm^3^)	Peak Gain (dBi)	Radiation Efficiency (%)	Coupling efficiency	Measurement repeatability	Operator experience requirement	Fabrication Cost	Material Cost	Eco-Friendly Material
^36^	Multi-angle Horn	1.7–2.1	N/A	31.7	80 +/− 15%	Medium	Repeatable	High	High	High	Non-biodegradable
^37^	Corrugated conical horn	0.211–0.275	25.0** × **29.0 × 20.0	26.5	N/A	High	Repeatable	High	High	High	Non-biodegradable
^38^	3D-printed back-to-back horn	0.22–0.325	10.2 × 43.25 × 10.2	~ 30	N/A	Medium	Repeatable	High	High	High	Non-biodegradable
^39^	Hemispherical lens antennas	0.22–0.32	6.0 × 18.8 × 6.0	16.1	N/A	Medium	Repeatable	High	Low	Low	Non-biodegradable
^40^	Integrated an *E*-plane flare and dual *H*-plane reflectors	0.325-0.5	N/A	32.0	43.75	Medium	Repeatable	High	Low	High	Non-biodegradable
^41^	Step-profiled corrugated horn antennas integrated in LTCC	0.242–0.33	5.0 × 5.0 × 2.8	18	90	Medium	Repeatable	High	High	Low	Non-biodegradable
^42^	*H*-plane corrugated horn antenna	0.191	5.0 × 5.0 × 0.75	9.8	N/A	Medium	Repeatable	High	High	High	Non-biodegradable
^43^	Wideband circularly polarized antennas	0.22–0.32	1.5 × 10.0 × 6.7	~ 13.5	98	Medium	Repeatable	High	High	High	Non-biodegradable
^44^	Metallic lens antenna	0.37–0.46	28.0 × 30.0 × 20.0	27.6	97	Medium	Repeatable	High	Low	High	Non-biodegradable
*This work*	*All-photopolymer Bragg Horn Antenna*	*0.22–0.325*	*55.6 × 55.6 × 203*	*28.98*	*98*	*High*	*Highly Repeatable*	*Low*	*Low*	*Low*	*Biodegradable*

The results indicate that the radiation pattern shows a strong correlation between the simulated and measured data within the angular range of − 30° to + 30°. This agreement highlights the accuracy of the antenna design and measurement setup in capturing the intended performance characteristics. However, beyond ± 30°, a noticeable mismatch is observed between the simulated and measured results. This discrepancy is attributed to significant free-space path loss, which reduces the dynamic range of the VNA and impacts the accuracy of measurements in these angular regions.

The antenna design presented in this work strikes an effective balance between achieving high gain and maintaining a compact form factor, while carefully accounting for the material loss characteristics of the chosen photopolymer. Although higher realized gains could potentially be obtained using lower-loss materials such as high-resistivity silicon or GaAs, these alternatives come with significantly higher costs and require complex manufacturing processes, such as those involving advanced cleanroom facilities. In contrast, the additive manufacturing approach employed in this study, particularly the 3D-printing technique, offers a simpler, more accessible fabrication method that is well-suited for rapid prototyping and mass production at substantially reduced costs. This approach not only simplifies the production process but also aligns with the growing demand for eco-friendly and cost-effective solutions in terahertz (THz) technology. Table 3 provides a detailed comparison of eight key performance parameters—including structure, coupling efficiency, repeatability, user expertise requirements, fabrication and material costs, and environmental impact—between the THz antenna proposed in this work and other state-of-the-art designs reported in the literature^36–44^. In addition, antenna size, peak gain, and radiation efficiency have been included to enable a more comprehensive performance evaluation. However, direct comparison of antenna size may not fully reflect performance differences, as the present work focuses on an all-dielectric photopolymer-based horn antenna that does not require high-precision milling machines. This approach significantly reduces fabrication complexity and cost, aligning with the goals of eco-friendly and accessible THz system development.

## Discussion and limitations

### Performance limitations of all-dielectric Horn antenna

While theoretical estimates based on beamwidth suggest higher directivity, the measured gain of the 3D-printed dielectric horn antenna reflects practical limitations not captured by idealized models. In particular, additive manufacturing processes introduce surface roughness, dimensional tolerances, and material inhomogeneities that can degrade radiation efficiency. The use of photopolymer materials further contributes to dielectric losses and scattering, especially at higher terahertz frequencies. Unlike metallic horns, which benefit from high conductivity and well-established machining tolerances, 3D-printed dielectric structures are subject to fabrication and material constraints that limit their ability to achieve theoretical peak performance. As a result, the realized gain and beam quality of the printed antenna may not reach the levels typically observed in precision-fabricated metallic counterparts. Nonetheless, the presented design offers a compelling balance between performance, manufacturability, and sustainability for next-generation terahertz systems.

### Experimental setup constraints and far-field measurement validity

The experimental validation of the radiation pattern was constrained by the physical setup of the laboratory. Although the far-field distance for the proposed Bragg fiber horn antenna, based on its aperture size, is estimated to be approximately 6.7 m, the measurement was performed at a distance of 50 cm. This distance was chosen to ensure practical feasibility, as it exceeds both the reactive near-field boundary of the Bragg horn (~ 0.27 m) and the far-field distance required for the standard horn antenna used as the transmitter (~ 67.9 mm). Extending the measurement distance to the full theoretical far-field range was not possible due to space constraints and excessive free-space path loss, which exceeds − 99 dB at such distances and limits signal detectability with the VNA. Despite this, the measured radiation patterns remained stable and closely matched simulation results, supporting the reliability of the data obtained under these conditions.

While the simulated cross-polarization levels and radiation pattern symmetry are presented in Figs. 3 and 4, the measurement of cross-polarization characteristics could not be performed due to current resource limitations. The polarization measurement setup, including the rotating positioner and VNA extensions, remains at the University of Leeds, whereas the authors have returned to Thailand. Nevertheless, full-wave simulations confirm that cross-polarization levels remain below − 35 dB across the main lobe in both principal planes, and the radiation patterns exhibit strong symmetry, providing meaningful insight into the antenna’s polarization purity.

### Environmental durability considerations

In addition to electromagnetic performance, the long-term durability of the proposed all-photopolymer Bragg horn antenna under environmental conditions is an important factor for practical deployment. The photopolymer material used in this study (Accura ClearVue) was selected for its optical clarity and compatibility with high-resolution additive manufacturing. According to the manufacturer’s datasheet and supplementary in-house accelerated aging tests, the antenna structure maintains mechanical stability and functional performance when exposed to moderate environmental conditions, including temperature cycles ranging from − 10 °C to 60 °C, relative humidity levels up to 85%, and UV exposure for durations up to 72 h. Minimal detuning of the resonance frequency and negligible structural deformation were observed under these conditions. However, for outdoor or long-term use in harsh environments, potential degradation due to UV-induced polymer breakdown or moisture absorption must be considered. Future work will explore protective surface coatings, encapsulation methods, or hybrid material integration (e.g., polymer-ceramic composites) to enhance environmental robustness while maintaining the advantages of low-cost additive fabrication.

### Scalability and fabrication repeatability

Scalability and manufacturing repeatability are critical for the practical deployment of the proposed 3D-printed Bragg fiber horn antenna. The antenna design was parametrically modeled to support scalability across different terahertz bands. Specifically, the flare angle, aperture size, and horn length are defined by frequency-dependent equations, allowing the design script to be readily scaled based on desired operating frequency and waveguide dimensions. This enables straightforward adaptation to bands such as WR-2.2 or WR-5 without requiring manual redesign of the structure.

To assess fabrication repeatability, three identical antenna prototypes were fabricated using the same SLA-based 3D printing process with Accura ClearVue photopolymer. Post-fabrication dimensional inspections revealed that critical geometrical features—such as aperture diameter, flare profile, and waveguide interface—were consistent within ± 3%^45^ tolerance. These results confirm that the photopolymer-based printing process offers reliable reproducibility for small-scale production, with minimal performance deviation among units. For larger-scale manufacturing, further work will explore printer calibration protocols, batch-to-batch material stability, and automated post-processing to maintain consistency.

## Conclusions

This study introduces the all-photopolymer Bragg horn antenna, specifically designed to operate within the WR-3.4 band, covering the 220–325 GHz frequency range. To evaluate its performance, three flare horn angles—5°, 15°, and 30°—were analyzed and compared based on key parameters, including the reflection coefficient (S_11_), realized gain, and half-power beamwidth (HPBW). To address coupling challenges resulting from the mismatch between the WR-3.4 aperture and the Bragg fiber aperture, a horn-type adapter was employed. This adapter facilitates the transition by connecting the standard WR-3.4 waveguide to the Bragg fiber input aperture, converting the TE_10_ mode from the rectangular waveguide into the fundamental HE_11_ mode within the Bragg fiber. Experimental results demonstrate that the all-photopolymer Bragg horn antenna achieves a fractional bandwidth of 38.5%, effectively covering the entire WR-3.4 band. The antenna exhibits a measured peak gain of approximately 28.98 dBi at 0°, a return loss better than − 20 dB across the operating band, and a consistent HPBW of approximately 5° throughout the WR-3.4 frequency range. The larger volume of the all-photopolymer Bragg horn antenna results from its integrated Bragg fiber structure, which is intentionally designed to ease connection and design while supporting efficient hybrid mode propagation and reducing dielectric and conductor losses across the WR-3.4 band, thus enabling low-cost fabrication through 3D printing. This design approach is inherently scalable and may be extended to higher-frequency bands or adapted for other terahertz antenna types, with potential integration into compact THz systems for imaging, sensing, and high-speed communication applications. Future work will explore co-integration with CMOS-compatible platforms, dielectric waveguides, and compact packaging techniques, as well as material improvements to enhance long-term environmental stability for real-world deployment.

## Data Availability

The datasets generated and/or analyzed during the current study are not publiclyavailable due to the following reasons: (i) the data forms part of ongoing research and is subject to further analysis in subsequent publications, (ii) certain aspects of the data are protected under institutional and collaborative agreements, and (iii) the dataset contains proprietary design files and fabrication parameters considered as intellectual property. However, relevant summary data supporting the findings of this study are included within the article and its supplementary information. Further information can be made available from the corresponding author upon reasonable request and subject to appropriate review and agreements.
